# Uncovering a Causal Connection between Gut Microbiota and Six Thyroid Diseases: A Two-Sample Mendelian Randomization Study

**DOI:** 10.3390/biology13090714

**Published:** 2024-09-11

**Authors:** Jiahao Chen, Yu Wang, Hang Yao, Yuxin Li, Hong Song

**Affiliations:** 1 School of Basic Medical Sciences, Zhejiang Chinese Medical University, Hangzhou 310053, China; jiahao0017@163.com; 2 Graduate School of Jiangxi, University of Chinese Medicine, Nanchang 330004, China; wangyudpk@163.com (Y.W.); rongyu1107@163.com (Y.L.); 3 School of Traditional Chinese Medicine, Binzhou Medical University, Yantai 264003, China; hanghang9703@163.com

**Keywords:** gut microbiota, causal relationship, thyroid diseases, mendelian randomization analysis, gut-thyroid axis

## Abstract

**Simple Summary:**

Thyroid diseases, such as goiter and thyroid nodules, are prevalent and can significantly impact people’s health and quality of life. Recent studies have indicated that the microorganisms in our gut, known as the gut microbiota, might influence the development of these conditions. However, whether these microorganisms cause thyroid diseases or are merely associated with them remains unclear. In this study, we employed genetic analysis to determine whether specific gut bacteria affect the risk of developing six common thyroid disorders. Our findings demonstrate that certain gut bacteria are indeed associated with these diseases—some bacteria seem to protect against thyroid conditions, while others may elevate the risk. This discovery highlights the important role that gut health plays in thyroid disease and suggests new possibilities for prevention and treatment. By targeting gut bacteria through dietary changes, probiotics, or other interventions, it may be feasible to reduce the risk of developing thyroid diseases. Our study underscores the vital connection between gut health and thyroid function, and its potential impact on overall well-being.

**Abstract:**

Background: Recent studies have established associations between the gut microbiota (GM) and thyroid diseases (TDs). However, their causal relationships remain elusive. Methods: To investigate this causality, we conducted a two-sample Mendelian randomization (MR) analysis using genome-wide association study (GWAS) data from MiBioGen and FinnGen, with GM as the exposure and six TDs as outcomes. Results: We identified 32 microbial taxa linked to the risk of six TDs. The *Clostridium innocuum group*, *Ruminiclostridium5*, and *Lachnoclostridium* exhibited protective effects against nontoxic diffuse goiter (NDG). Conversely, an increased risk of NDG was associated with *Ruminococcaceae UCG002*, *Alistipes*, *Methanobrevibacter*, *Marvinbryantia*, and *Ruminococcaceae UCG014*. *Bifidobacterium* and *Sutterella* were protective against nontoxic multinodular goiter (NMG), while the *Ruminococcus gauvreauii group* and *Rikenellaceae RC9 gut group* heightened NMG risk. Protective effects against nontoxic single thyroid nodule (NSTN) were observed with *Defluviitaleaceae UCG011*, *Ruminococcus1*, and *Ruminococcaceae UCG010*, whereas increased risk was linked to *Alistipes*, the *Ruminococcus gauvreauii group*, and *Lachnospiraceae UCG010*. *Ruminiclostridium9*, *Victivallis*, and *Butyricimonas* offered protection against thyrotoxicosis with Graves’ Disease (GD), while the *Eubacterium rectale group*, *Desulfovibrio*, *Bifidobacterium*, *Collinsella*, *Oscillospira*, and *Catenibacterium* were risk factors. For thyrotoxicosis with Plummer Disease (PD), protective taxa included *Butyricimonas* and *Lachnospira*, whereas *Dorea*, *Eggerthella*, *Odoribacter*, *Lactobacillus*, *Intestinimonas*, and *Phascolarctobacterium* increased risk. Lastly, *Parasutterella* was protective against thyrotoxicosis with toxic single thyroid nodule (TSTN), while increased risk was associated with *Sutterella*, *Oscillibacter*, and *Clostridium sensu stricto1*. Conclusions: Our findings support a causal relationship between specific GM and TDs at the genetic level, laying the foundation for future research into potential mechanisms and the identification of novel therapeutic targets.

## 1. Introduction

Thyroid diseases (TDs) encompass various disorders of the thyroid gland, including nontoxic diffuse goiter (NDG), nontoxic multinodular goiter (NMG), nontoxic single thyroid nodule (NSTN), Graves’ disease (GD), Plummer disease (PD), and thyrotoxicosis with toxic single thyroid nodule (TSTN). These conditions are prevalent organ-specific disorders. Their etiology may be linked to congenital factors, insufficient iodine intake, or autoimmune responses [1,2,3]. Recent data indicate that TDs affect approximately 5% of the general population, with a higher incidence in females [4]. These conditions significantly influence morbidity and quality of life [5]. Although the pathogenesis of these diseases is not fully elucidated, it is thought to be influenced by a combination of genetic and environmental factors.

The gut microbiota (GM) comprises the microbial community within the human gut, forming a complex microecosystem, particularly within the gastrointestinal tract [6]. It is estimated that the human gut hosts about 1000 species of microbes, constituting 78% of the total microbial cell count in the body [7]. The GM is responsible for various regulatory functions, such as maintaining the integrity of the gut mucosal barrier and modulating the immune system [6]. Disruptions in this microbial balance can compromise the host’s normal physiological functions, leading to various diseases. The link between the GM and the thyroid is referred to as the “gut-thyroid axis” [8]. Previous studies have demonstrated that the GM and its metabolites may influence thyroid function through the modulation of trace element absorption in the gut and through inflammatory and immune responses [9].

Mendelian randomization (MR) is a genetics-based method crucial for exploring the causal connections between the GM and TDs [10]. In the absence of feasible randomized controlled trials, MR has emerged as a significant alternative for assessing the causal relationship between theGM and disease risk. Its primary advantage lies in using naturally occurring genetic variations that are assigned randomly during fertilization, thereby minimizing confounding in causal analysis, and remaining largely unaffected by the disease process. Thus, it facilitates a robust assessment of the causal relationships between the GM and various TDs. MR has previously established a causal relationship between genetically predicted GM and conditions such as thyroid cancer [11], hypothyroidism [12], and Hashimoto’s thyroiditis [13]. However, the causal role of the GM in many other TDs has not yet been reported.

To identify potential GM links to the etiology of TDs, we utilized the most recent and comprehensive GWAS summary data for a two-sample MR analysis. This investigation advances our understanding of the pathogenesis of TDs by exploring the causal connections between the human GM and TDs.

## 2. Materials and Methods

### 2.1. Study Overview

**Figure 1:**
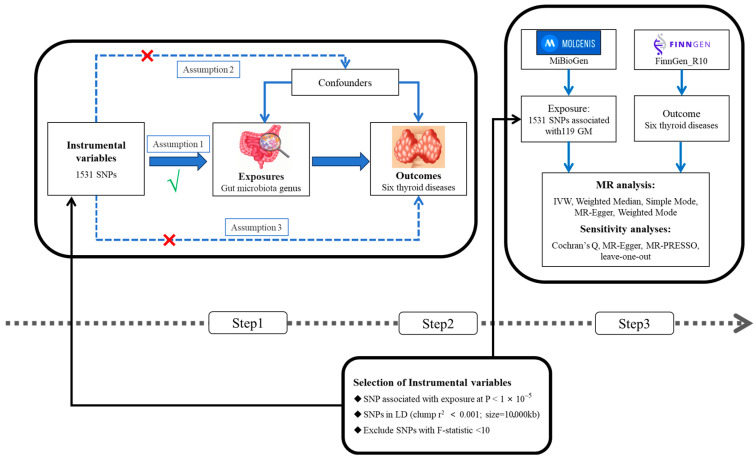
(Step 1) Assumptions of the study. (Step 2) Selection of instrumental variables. (Step 3) Analytical process.

This study employed a two-sample MR analysis to assess the causal relationship between the GM and six TDs. GWAS summary-level data for the GM and TDs were acquired separately, treating each genus-level GM as an independent exposure, and grouping four thyroid nodules and two solitary nodules as outcomes. For accurate results, this MR analysis must meet three criteria: (1) the selected instrumental variables (IVs) must demonstrate a strong association with the GM; (2) the IVs must not be associated with any potential confounding factors; (3) the IVs should influence the risk of TDs solely through the GM, without any other intervening mechanisms [14,15]. Figure 1 illustrates the analytical process.

### 2.2. Data Sources

Initially, GWAS data for the GM were sourced from the MiBioGen consortium, which involved 18,340 participants [16]. Twelve unknown genera were excluded, resulting in 119 genera included for analysis [17]. GWAS data for various TDs, including NDG, NMG, NSTN, GD, PD, and TSTN, were sourced from the FinnGen R10 database [18], with all data deriving from European populations. The summary data utilized in this study were obtained from public databases, and each GWAS had received ethical approval from its respective institution, as detailed in Table 1.

### 2.3. Selection of Instrumental Variables

Initially, single nucleotide polymorphisms (SNPs) strongly associated with specific genera were selected as IVs using a significance threshold (*p* < 1 × 10^−5^). Next, linkage disequilibrium (LD) analysis was performed using European genome sample data, with parameters set to kb = 10,000 and r^2^ < 0.001 [19], while excluding palindromic SNPs to prevent allelic effects on the outcomes. Finally, the strength of the IVs was evaluated by calculating the F-statistic; an F-value greater than 10 indicates the absence of weak instrument bias, and IVs with an F ≤ 10 were excluded. The F-value is calculated as follows: F = β^2^ exposure/SE^2^ exposure [17].

### 2.4. Mendelian Randomization Analysis and Sensitivity Analysis

This study conducted a comprehensive assessment of the potential causal links between the GM and six TDs using five different MR methods: inverse variance weighted (IVW), weighted median, simple mode, MR-Egger, and weighted mode, with IVW as the primary method [20].

Cochran’s Q test was used to evaluate the heterogeneity of the results, with a *p*-value greater than 0.05 indicating no heterogeneity. Horizontal pleiotropy was assessed using MR-Egger and MR-PRESSO tests, where an intercept *p*-value above 0.05 suggested no horizontal pleiotropy. A leave-one-out analysis was conducted to determine the impact of any single SNP on the study results, and funnel plots and forest plots were utilized for visualization to ensure the robustness of the findings [21]. The analysis was performed using R-4.3.2 software.

## 3. Results

### 3.1. Instrumental Variables and Mendelian Randomization Results

**Figure 2:**
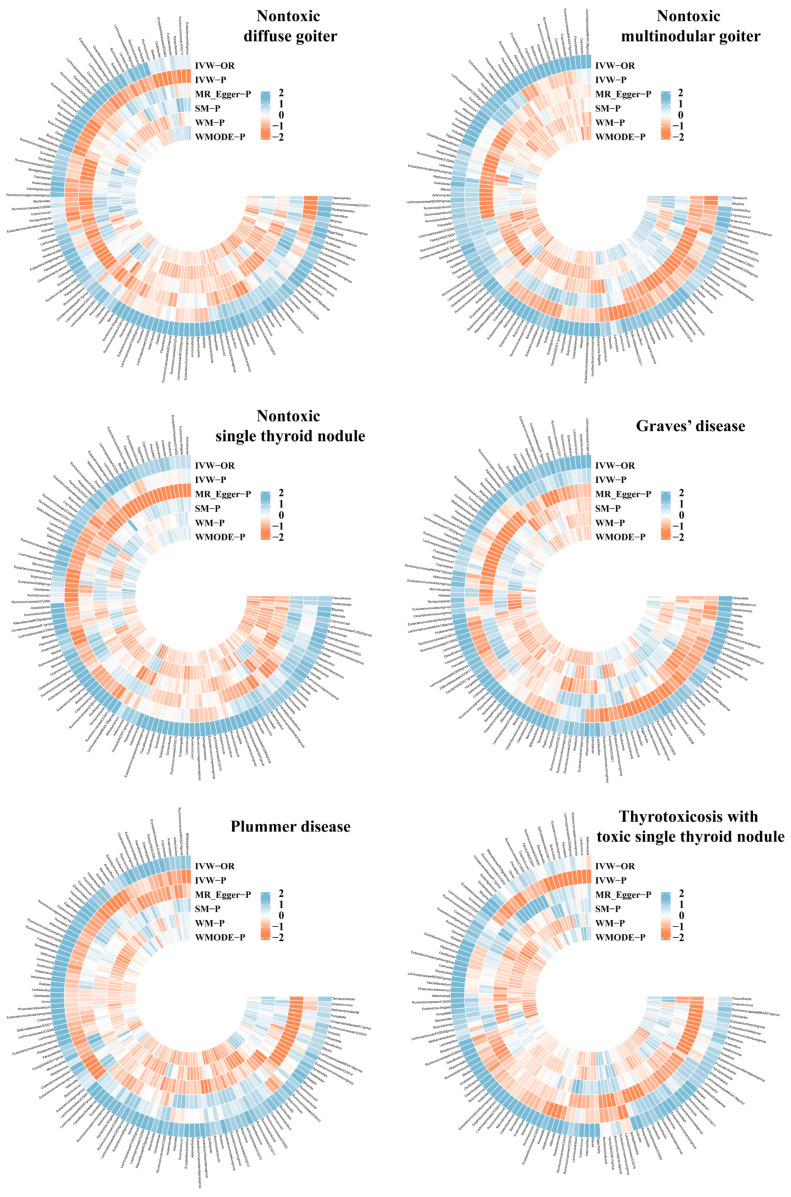
Circus plot showing the MR results for all GM.

Following the selection criteria, a total of 1531 SNPs from 119 GM genera were identified as IVs [22], all exhibiting an F-statistic greater than 10. This confirms that our study is not susceptible to weak instrument bias, with details provided in Supplementary Table S1. The MR analysis results for each of the 119 genera in relation to the six TDs are displayed in Figure 2 and Supplementary Tables S2–S7.

### 3.2. Causal Relationships between Gut Microbiota and Thyroid Diseases

**Figure 3:**
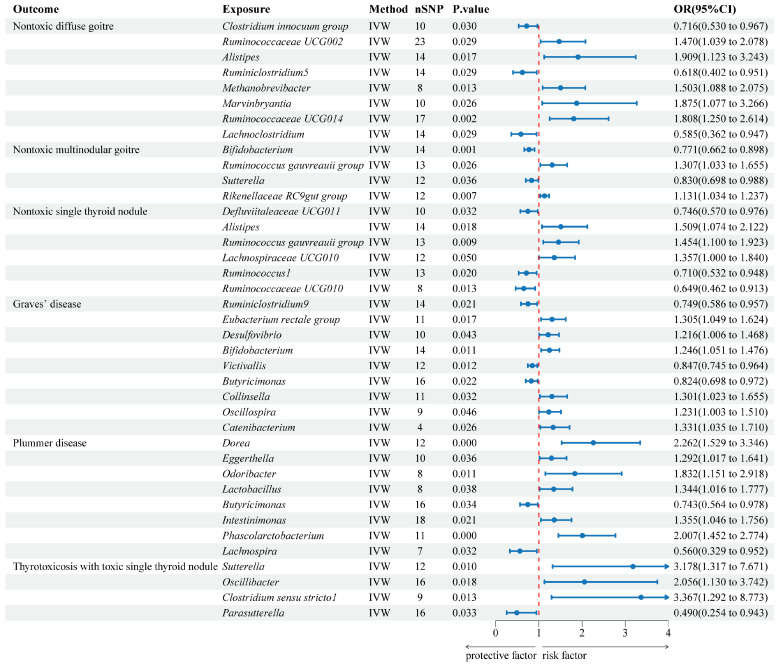
Forest plot of the associations between genetically predicted GM and 6 types of TDs risk using IVW methods.

The results from the IVW analysis revealed that 32 genera in the GM are associated with a risk of six TDs. Specifically, the *Clostridium innocuum group* (OR = 0.716, 95% CI: 0.530–0.967), *Ruminiclostridium5* (OR = 0.618, 95% CI: 0.402–0.951), and *Lachnoclostridium* (OR = 0.585, 95% CI: 0.362–0.947) were negatively associated with the risk of NDG. Conversely, *Ruminococcaceae UCG002* (OR = 1.470, 95% CI: 1.039–2.078), *Alistipes* (OR = 1.909, 95% CI: 1.123–3.243), *Methanobrevibacter* (OR = 1.503, 95% CI: 1.088–2.075), *Marvinbryantia* (OR = 1.875, 95% CI: 1.077–3.266), and *Ruminococcaceae UCG014* (OR = 1.808, 95% CI: 1.250–2.614) were associated with increased NDG risk. *Bifidobacterium* (OR = 0.771, 95% CI: 0.662–0.898) and *Sutterella* (OR = 0.830, 95% CI: 0.698–0.988) provided protection against NMG, while the *Ruminococcus gauvreauii group* (OR = 1.307, 95% CI: 1.033–1.655) and *Rikenellaceae RC9 gut group* (OR = 1.131, 95% CI: 1.034–1.237) increased NMG risk. *Defluviitaleaceae UCG011* (OR = 0.746, 95% CI: 0.570–0.976), *Ruminococcus1* (OR = 0.710, 95% CI: 0.532–0.948), and *Ruminococcaceae UCG010* (OR = 0.649, 95% CI: 0.462–0.913) had protective effects against NSTN, while *Alistipes* (OR = 1.509, 95% CI: 1.074–2.122), the *Ruminococcus gauvreauii group* (OR = 1.454, 95% CI: 1.100–1.923), and *Lachnospiraceae UCG010* (OR = 1.357, 95% CI: 1.000–1.840) were associated with increased NSTN risk. *Ruminiclostridium9* (OR = 0.749, 95% CI: 0.586–0.957), *Victivallis* (OR = 0.847, 95% CI: 0.745–0.964), and *Butyricimonas* (OR = 0.824, 95% CI: 0.698–0.972) were negatively associated with the risk of GD, while the *Eubacterium rectale group* (OR = 1.305, 95% CI: 1.049–1.624), *Desulfovibrio* (OR = 1.216, 95% CI: 1.006–1.468), *Bifidobacterium* (OR = 1.246, 95% CI: 1.051–1.476), *Collinsella* (OR = 1.301, 95% CI: 1.023–1.655), *Oscillospira* (OR = 1.231, 95% CI: 1.003–1.510), and *Catenibacterium* (OR = 1.331, 95% CI: 1.035–1.710) increased GD risk. *Butyricimonas* (OR = 0.743, 95% CI: 0.564–0.978) and *Lachnospira* (OR = 0.560, 95% CI: 0.329–0.952) provided protection against PD, while *Dorea* (OR = 2.262, 95% CI: 1.529–3.346), *Eggerthella* (OR = 1.292, 95% CI: 1.017–1.641), *Odoribacter* (OR = 1.832, 95% CI: 1.151–2.918), *Lactobacillus* (OR = 1.344, 95% CI: 1.016–1.777), *Intestinimonas* (OR = 1.355, 95% CI: 1.046–1.756), and *Phascolarctobacterium* (OR = 2.007, 95% CI: 1.452–2.774) increased PD risk. *Parasutterella* (OR = 0.490, 95% CI: 0.254–0.943) was negatively associated with the risk of TSTN, while *Sutterella* (OR = 3.178, 95% CI: 1.317–7.671), *Oscillibacter* (OR = 2.056, 95% CI: 1.130–3.742), and *Clostridium sensu stricto1* (OR = 3.367, 95% CI: 1.292–8.773) increased TSTN risk. These findings are visually represented in Figure 3 and the scatter plots in Supplementary Figure S1.

### 3.3. Sensitivity Analysis

In the NMG, the *Ruminococcus gauvreauii group* displayed heterogeneity and horizontal pleiotropy (Cochran’s Q *p*-value = 0.014, MR-PRESSO *p*-value = 0.024), potentially impacting the final study results. After the outliers rs2047242 and rs289410 were removed, a subsequent sensitivity analysis was conducted. The revised results indicate that the *Ruminococcus gauvreauii group* no longer exhibited heterogeneity or horizontal pleiotropy (Cochran’s Q *p*-value = 0.503, MR-PRESSO *p*-value = 0.567). Horizontal pleiotropy was absent in other genera, as documented in Table 2. The leave-one-out analysis, along with funnel plots and forest plots, did not identify any significant outliers, confirming the reliability of the MR study findings, as illustrated in Supplementary Figures S2–S4.

## 4. Discussion

Numerous studies have indicated that dysbiosis within the GM may precipitate various TDs [23,24,25]. However, definitively ascertaining a causal link between GM and TDs is problematic due to inherent limitations in both observational and experimental methodologies. Recent advances in sequencing technologies have shed light on the integral role of GM in disease regulation, encompassing immune function, metabolic processes, and pharmacokinetics. Utilizing the concept of the gut–thyroid axis, researchers have begun to investigate how GM affect thyroid activity through their impact on micronutrient absorption and immune modulation.

The present research is pioneering in its use of the MR approach to explore causal relationships between GM and the incidence of six distinct TDs. The GM, characterized as a dynamic and multifaceted ecosystem, represents a novel area of inquiry [26]. Our results suggest that specific microbial taxa are causally linked to the prevalence of these TDs.

This study also delineates the intricate genetic interactions contributing to the emergence of TDs, which frequently manifest as chronic thyroid enlargement and are commonly referred to as goiter. Goiters are classified into NDG and NMG, along with hyperthyroid conditions such as GD and multiple PD. At the genus level, we identified 32 GM taxa correlated with these conditions. Notably, the *Clostridium innocuum group*, *Ruminiclostridium5*, and *Lachnoclostridium* appear to exert protective effects against NDG. Conversely, taxa such as *Ruminococcaceae UCG002*, *Alistipes*, *Methanobrevibacter*, *Marvinbryantia*, and *Ruminococcaceae UCG014* elevate NDG risk. For NMG, *Bifidobacterium* and *Sutterella* have been found to protect against it, whereas the *Ruminococcus gauvreauii group* and *Rikenellaceae RC9 gut group* increase NMG susceptibility. Protective agents for GD include *Ruminiclostridium9*, *Victivallis*, and *Butyricimonas*, while taxa such as the *Eubacterium rectale group*, *Desulfovibrio*, *Bifidobacterium*, *Collinsella*, *Oscillospira*, and *Catenibacterium* are linked to a heightened risk of GD. Furthermore, *Butyricimonas* and *Lachnospira* offer protection against PD, while *Dorea*, *Eggerthella*, *Odoribacter*, *Lactobacillus*, *Intestinimonas*, and *Phascolarctobacterium* are associated with increased PD risk.

The GM influences thyroid health through mechanisms such as immune regulation, anti-inflammatory responses, and increased intestinal permeability. It is essential to highlight that *Bifidobacterium*, prevalent in both human and animal guts and part of the Firmicutes phylum, is crucial for maintaining intestinal health and immune balance. Nonetheless, its association with TDs, especially NMG and GD, involves intricate mechanisms tied to inflammatory responses and immune regulation. Our findings indicate that *Bifidobacterium* provides protection against NMG—likely via its metabolic byproducts, such as short-chain fatty acids—which could reduce intestinal inflammation and autoimmunity, thus potentially decreasing NMG risk [27,28,29]. Conversely, evidence from this study suggests there is an increased risk of GD associated with *Bifidobacterium*, challenging prevailing views. GM might play a critical role in GD’s pathogenesis [30]. For example, research shows that combining *Bifidobacterium* with methimazole may affect neurotransmitters and blood micronutrients through the gut–brain and gut–thyroid axes, thereby enhancing thyroid function in GD patients [31].

Furthermore, while the roles of some microbial taxa in specific TDs remain unreported, *Lachnoclostridium*—an anaerobic, Gram-positive bacterium from the Firmicutes phylum—is known to offer protective effects against NDG. This protection may be attributed to its production of anti-inflammatory butyrate salts and the upregulation of tight junction proteins, thus enhancing the gut barrier [32]. Similarly, *Sutterella*, a genus of Gram-negative bacteria from the Bacteroidota phylum, appears to provide protection against NMG, possibly by adhering to intestinal cells and modulating immune responses [33,34]. Both GD and PD lead to excessive hormone production by the thyroid, resulting in hyperthyroidism and thyroid enlargement. GD typically manifests as diffuse thyroid enlargement, while PD features multiple nodular enlargements [35,36]. Studies have indicated significantly lower levels of *Butyricimonas* in thyroid nodules [37]. Further research into the relationship between the gut microbiome, its metabolites, and thyroid nodules reveals a marked reduction in *Butyricimonas* in patients with thyroid nodules and thyroid cancer [9]. Research in hyperthyroid gerbils has shown diminished levels of beneficial *Butyricimonas*, which is involved in regulating the host’s resting metabolic rate and food intake, suggesting that thyroid hormones might alter host thermogenesis by modifying the gut microbiome [38]. Our findings corroborate these observations, showing that *Butyricimonas* also offers protection against GD and PD. NSTN and thyrotoxicosis with TSTN both involve a single thyroid nodule, with the primary distinction being whether the nodule induces hyperthyroidism. An NSTN is generally asymptomatic, whereas a TSTN leads to symptoms of hyperthyroidism. Additionally, our study expanded the analysis to include NSTN and TSTN. Protective taxa against NSTN include *Defluviitaleaceae UCG011*, *Ruminococcus1*, and *Ruminococcaceae UCG010*, while taxa such as *Alistipes*, the *Ruminococcus gauvreauii group*, and *Lachnospiraceae UCG010* are associated with an increased risk of NSTN. *Parasutterella* offers protection against TSTN, whereas *Sutterella*, *Oscillibacter*, and *Clostridium sensu stricto1* are linked to a higher TSTN risk. Notably, *Alistipes*, a Gram-negative anaerobic bacterium from the Rikenellaceae family within the *Bacteroidetes* phylum, is identified as a pathogenic microbial modulator and is associated with an increased risk of NDG and NSTN [39]. Continued research is necessary to define the specific mechanisms through which other microbial taxa impact TDs.

## 5. Conclusions

In summary, our research has established a causal link between the GM and six TDs. However, several inherent limitations in our study must be acknowledged. First, the dataset was limited to a European population, which may not be representative of other ethnic groups. Second, the analysis of the GM was conducted at the genus level, rather than at a more detailed or specialized level. Third, our analysis did not address gender differences in the relationship between GM and the six types of TDs. In future studies, gender will be considered as a potential factor. Finally, the reliance on publicly available GWAS data may introduce various biases and limitations that warrant careful consideration. Further in-depth analysis will be required to address these issues and enhance our understanding.

## Figures and Tables

**Table 1:** Sources of data for the study.

Trait	Data Type	N_Cases	N_Controls	Consortium	Phenocode
GM	Exposure	18,340		MiBioGen	
NDG	Outcome	906	349,717	FinnGen_R10	E4_GOITREDIFF
NMG	Outcome	6699	349,717	FinnGen_R10	E4_GOITREMULTINOD
NSTN	Outcome	2203	349,717	FinnGen_R10	E4_GOITRENOD
GD	Outcome	4846	349,717	FinnGen_R10	E4_THYTOXGOITDIF
PD	Outcome	1717	403,309	FinnGen_R10	E4_THYTOXGOITMULT
TSTN	Outcome	246	403,309	FinnGen_R10	E4_THYTOXNOD

**Table 2:** Sensitivity analysis results for this study.

Outcome	Exposure	Heterogeneity		Directional Pleiotropy		MR-PRESSO
		Cochran’s Q	*p*-Value	Egger Intercept	*p*-Value	*p*-Value
NDG	*Clostridium innocuum group*	9.361	0.313	−0.111	0.323	0.358
	*Ruminococcaceae UCG002*	20.528	0.488	−0.015	0.672	0.561
	*Alistipes*	6.909	0.864	0.082	0.323	0.853
	*Ruminiclostridium5*	8.642	0.733	−0.007	0.870	0.842
	*Methanobrevibacter*	2.917	0.819	−0.014	0.881	0.882
	*Marvinbryantia*	10.957	0.204	0.019	0.858	0.282
	*Ruminococcaceae UCG014*	12.087	0.672	0.012	0.775	0.786
	*Lachnoclostridium*	10.957	0.533	0.027	0.648	0.598
NMG	*Bifidobacterium*	12.431	0.412	0.019	0.271	0.421
	*Ruminococcus gauvreauii group*	23.652	0.014	−0.030	0.446	0.024
		8.311	0.503	−0.022	0.418	0.567
	*Sutterella*	4.991	0.892	−0.004	0.892	0.933
	*Rikenellaceae RC9 gut group*	3.956	0.949	−0.007	0.863	0.974
NSTN	*Defluviitaleaceae UCG011*	3.316	0.913	0.051	0.360	0.914
	*Alistipes*	10.944	0.534	−0.014	0.787	0.639
	*Ruminococcus gauvreauii group*	7.889	0.723	−0.027	0.547	0.778
	*Lachnospiraceae UCG010*	4.287	0.933	−0.006	0.875	0.965
	*Ruminococcus1*	6.486	0.839	0.021	0.524	0.865
	*Ruminococcaceae UCG010*	1.724	0.943	0.010	0.795	0.973
GD	*Ruminiclostridium9*	16.142	0.185	−0.027	0.540	0.239
	*Eubacterium rectale group*	8.752	0.461	−0.026	0.262	0.487
	*Desulfovibrio*	7.665	0.467	0.013	0.667	0.602
	*Bifidobacterium*	10.787	0.547	0.004	0.835	0.668
	*Victivallis*	14.821	0.139	0.065	0.281	0.138
	*Butyricimonas*	11.642	0.635	0.033	0.238	0.605
	*Collinsella*	6.405	0.699	0.003	0.924	0.781
	*Oscillospira*	7.463	0.382	0.002	0.958	0.520
	*Catenibacterium*	4.388	0.111	−0.177	0.501	0.225
PD	*Dorea*	6.328	0.787	−0.062	0.149	0.680
	*Eggerthella*	8.349	0.400	0.019	0.768	0.516
	*Odoribacter*	5.514	0.480	−0.085	0.155	0.351
	*Lactobacillus*	6.446	0.375	−0.050	0.267	0.382
	*Butyricimonas*	10.576	0.719	−0.058	0.213	0.695
	*Intestinimonas*	11.493	0.778	0.013	0.671	0.833
	*Phascolarctobacterium*	8.348	0.500	−0.015	0.793	0.659
	*Lachnospira*	4.236	0.516	0.133	0.192	0.389
TSTN	*Sutterella*	9.669	0.470	−0.006	0.965	0.591
	*Oscillibacter*	13.415	0.494	0.032	0.763	0.596
	*Clostridium sensu stricto1*	6.494	0.483	0.090	0.473	0.554
	*Parasutterella*	14.629	0.404	−0.003	0.968	0.504

## Data Availability

The data from this study have been deposited in online repositories. The repository/repositories and corresponding accession number(s) can be located in the article’s Supplementary Materials.

## Supplementary Materials

The following supporting information can be downloaded at: https://www.mdpi.com/article/10.3390/biology13090714/s1, Figure S1: Scatter plots of causal estimates of gut microbiota on thyroid diseases; Figure S2: Leave-one-out stability tests causal estimates of specific GM on thyroid diseases; Figure S3: Funnel plots for causal effects of specific GM on thyroid diseases risk with individual SNPs; Figure S4: Forest plots for causal effects of specific GM on thyroid diseases risk with individual SNPs; Table S1: Detailed information of instrumental variables used in MR analyses; Table S2: The MR results of the associations between GM with NDG; Table S3: The MR results of the associations between GM with NMG; Table S4: The MR results of the associations between GM with NSTN; Table S5: The MR results of the associations between GM with GD; Table S6: The MR results of the associations between GM with PD; Table S7: The MR results of the associations between GM with TSTN.
