# Supplementary material for: Uncovering a Causal Connection between Gut Microbiota and Six Thyroid Diseases: A Two-Sample Mendelian Randomization Study
Source: Biology (Basel). 2024 Sep 11;13(9):714. doi: 10.3390/biology13090714 (PMC11428278; doi:10.3390/biology13090714)

Supplementary Material

Supplementary Figure S1.

Scatter plots of causal estimates of gut microbiota on thyroid diseases. The slope of each line corresponding to the estimated MR effect in different models, including the conventional IVW, Weighted median, MR-Egger, Simple mode, and Weighted mode. A-H): Nontoxic diffuse goiter; I-L): Nontoxic multinodular goiter; M-R): Nontoxic single thyroid nodule; S-AA): Graves Disease; AB-AI): Plummer Disease; AJ-AM): Thyrotoxicosis with toxic single thyroid nodule.

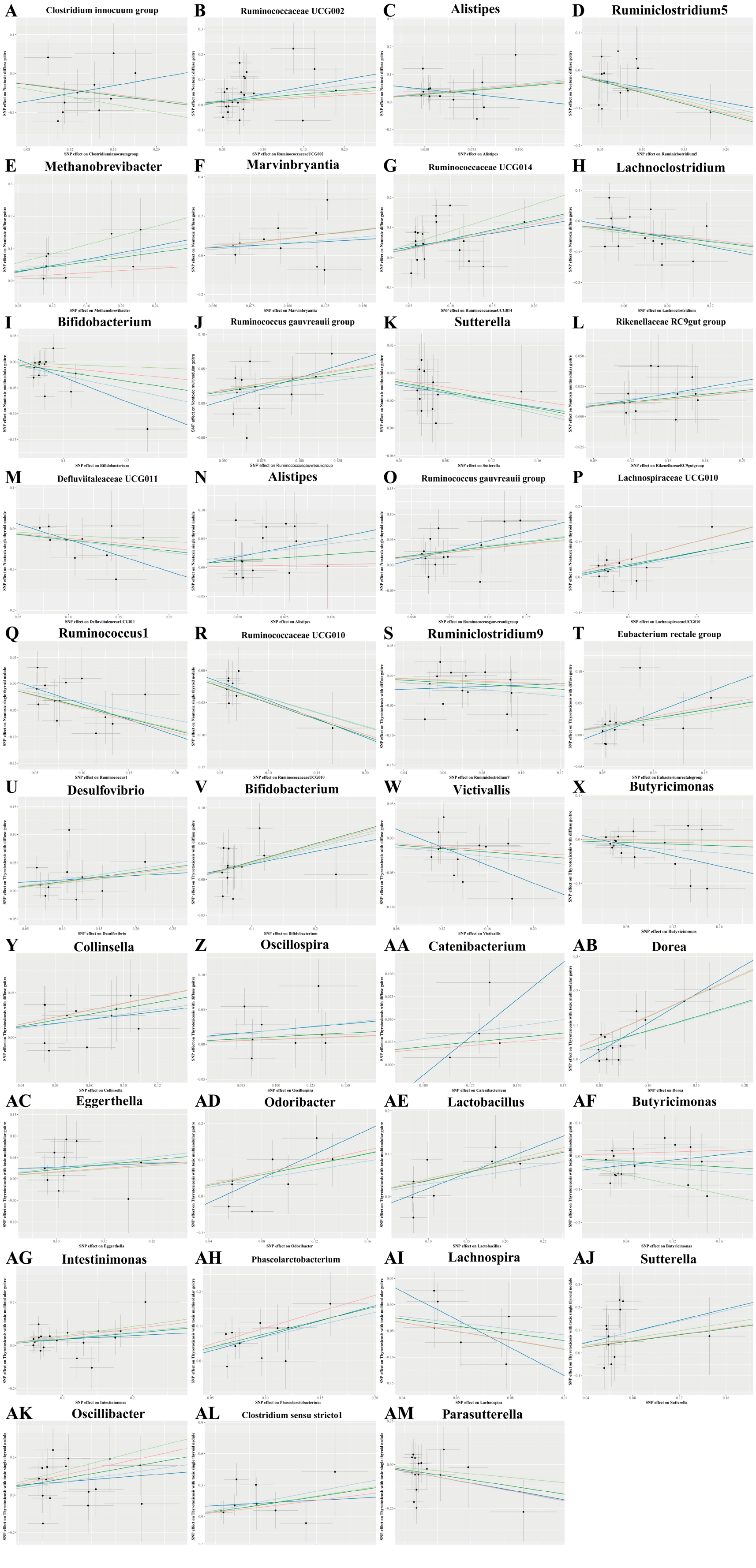

A Clostridium innocuum group
B Ruminococcaceae UCG002
C Alistipes
D Ruminiclostridium5
E Methanobrevibacter
F Marvinbryantia
G Ruminococcaceae UCG014
H Lachnoclostridium
I Bifidobacterium
J Ruminococcus gauvreauii group
K Sutterella
L Rikenellaceae RC9gut group
M Defluviitaleaceae UCG011
N Alistipes
O Ruminococcus gauvreauii group
P Lachnospiraceae UCG010
Q Ruminococcus1
R Ruminococcaceae UCG010
S Ruminiclostridium9
T Eubacterium rectale group
U Desulfovibrio
V Bifidobacterium
W Victivallis
X Butyricimonas
Y Collinsella
Z Oscillospira
AA Catenibacterium
AB Dorea
AC Eggerthella
AD Odoribacter
AE Lactobacillus
AF Butyricimonas
AG Intestinimonas
AH Phascolarctobacterium
AI Lachnospira
AJ Sutterella
AK Oscillibacter
AL Clostridium sensu stricto1
AM Parasutterella

Supplementary Figure S2. Leave-one-out stability tests causal estimates of specific GM on thyroid diseases. Calculate the MR results of the remaining IVs after removing the IVs one by one. A-H): Nontoxic diffuse goiter; I-L): Nontoxic multinodular goiter; M-R): Nontoxic single thyroid nodule; S-AA): Graves Disease; AB-AI): Plummer Disease; AJ-AM): Thyrotoxicosis with toxic single thyroid nodule.

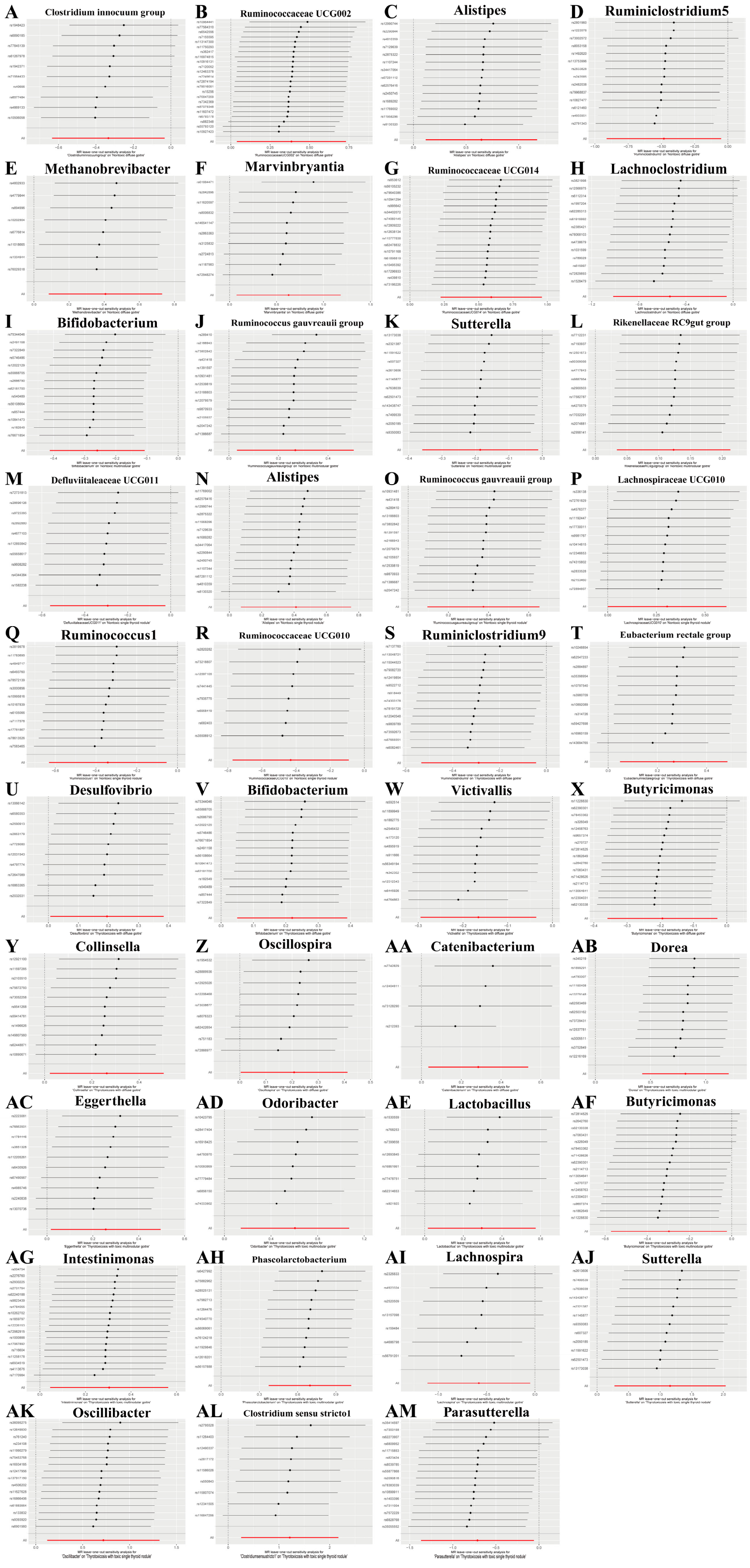
A Clostridium innocuum group
B Ruminococcaceae UCG002
C Alistipes
D Ruminiclostridium5
E Methanobrevibacter
F Marvinbryantia
G Ruminococcaceae UCG014
H Lachnoclostridium
I Bifidobacterium
J Ruminococcus gauvreauii group
K Sutterella
L Rikenellaceae RC9gut group
M Defluviitaleaceae UCG011
N Alistipes
O Ruminococcus gauvreauii group
P Lachnospiraceae UCG010
Q Ruminococcus1
R Ruminococcaceae UCG010
S Ruminiclostridium9
T Eubacterium rectale group
U Desulfovibrio
V Bifidobacterium
W Victivallis
X Butyricimonas
Y Collinsella
Z Oscillospira
AA Catenibacterium
AB Dorea
AC Eggerthella
AD Odoribacter
AE Lactobacillus
AF Butyricimonas
AG Intestinimonas
AH Phascolarctobacterium
AI Lachnospira
AJ Sutterella
AK Oscillibacter
AL Clostridium sensu stricto1
AM Parasutterella

Supplementary Figure S3. Funnel plots for causal effects of specific GM on thyroid diseases risk with individual SNPs. A-H): Nontoxic diffuse goiter; I-L): Nontoxic multinodular goiter; M-R): Nontoxic single thyroid nodule; S-AA): Graves Disease; AB-AI): Plummer Disease; AJ-AM): Thyrotoxicosis with toxic single thyroid nodule.

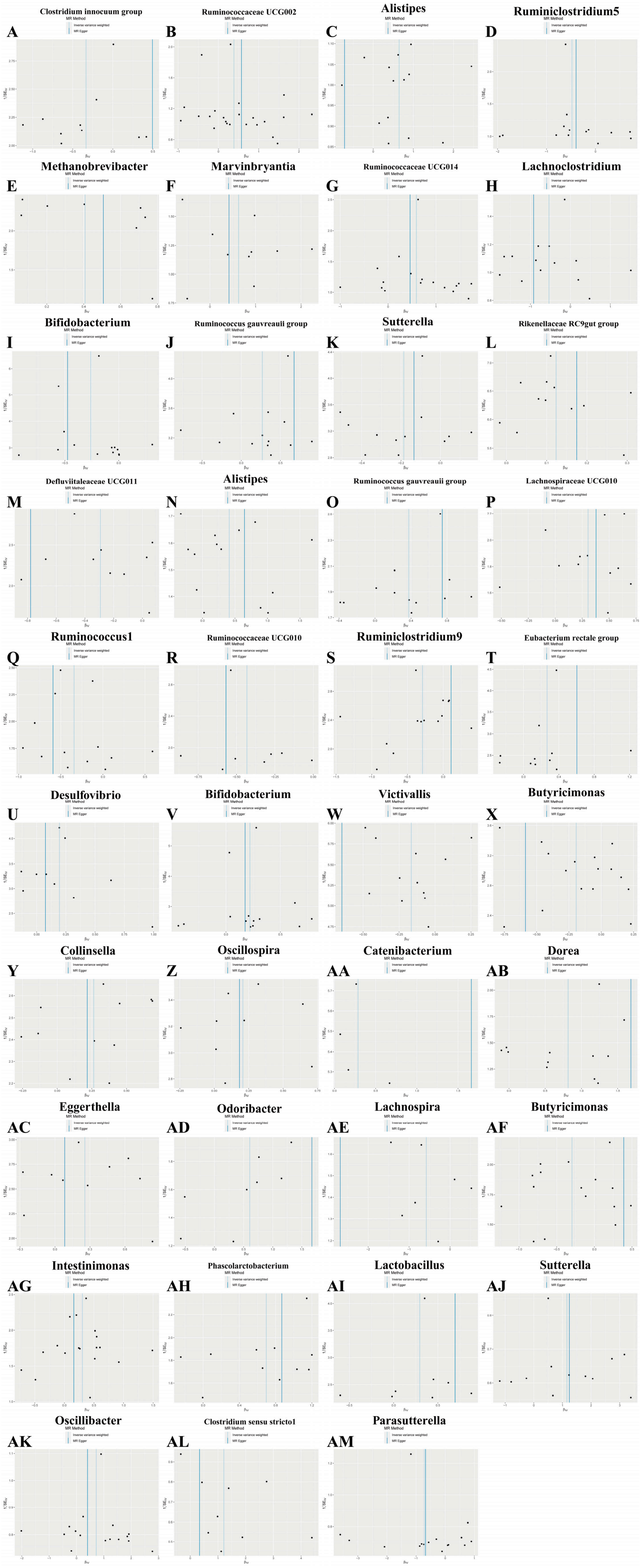

A Clostridium innocuum group
B Ruminococcaceae UCG002
C Alistipes
D Ruminiclostridium5
E Methanobrevibacter
F Marvinbryantia
G Ruminococcaceae UCG014
H Lachnoclostridium
I Bifidobacterium
J Ruminococcus gauvreauii group
K Sutterella
L Rikenellaceae RC9gut group
M Defluviitaleaceae UCG011
N Alistipes
O Ruminococcus gauvreauii group
P Lachnospiraceae UCG010
Q Ruminococcus1
R Ruminococcaceae UCG010
S Ruminiclostridium9
T Eubacterium rectale group
U Desulfovibrio
V Bifidobacterium
W Victivallis
X Butyricimonas
Y Collinsella
Z Oscillospira
AA Catenibacterium
AB Dorea
AC Eggerthella
AD Odoribacter
AE Lachnospira
AF Butyricimonas
AG Intestinimonas
AH Phascolarctobacterium
AI Lactobacillus
AJ Sutterella
AK Oscillibacter
AL Clostridium sensu stricto1
AM Parasutterella
MR Method
Inverse variance weighted
MR Egger

Supplementary Figure S4. Forest plots for causal effects of specific GM on thyroid diseases risk with individual SNPs. A-H): Nontoxic diffuse goiter; I-L): Nontoxic multinodular goiter; M-R): Nontoxic single thyroid nodule; S-AA): Graves Disease; AB-AI): Plummer Disease; AJ-AM): Thyrotoxicosis with toxic single thyroid nodule.

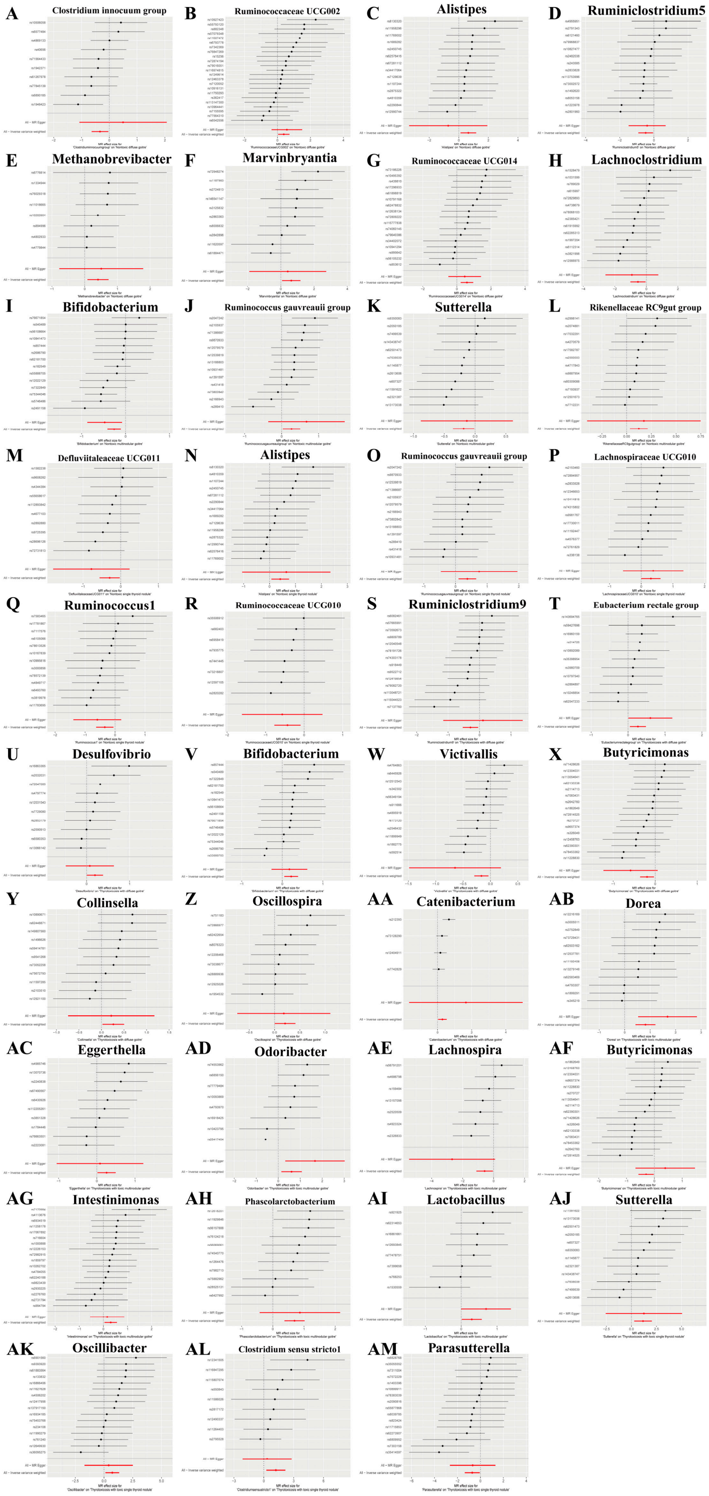

Supplement: Supplementary file 1 [file biology-13-00714-s001.zip › biology-3167710-supplementary/Supplementary Figures.pdf]
